# A qualitative exploration of the perceptions of Cardiff University medical students towards the barriers faced by the LGBTQ+ population when accessing mental health care

**DOI:** 10.1192/j.eurpsy.2025.2010

**Published:** 2025-08-26

**Authors:** E. Sammut, A. Hassoulas, S. Edney

**Affiliations:** 1 Mount Carmel Hospital, Attard, Malta; 2 Cardiff University, Cardiff, United Kingdom

## Abstract

**Introduction:**

Several mental disorders are more common within the LGBTQ+ population, including depression, anxiety, eating disorders, and substance use disorders. Despite this, LGBTQ+ individuals face more barriers when accessing mental health services than the general population.

**Objectives:**

The primary aim of this study was to assess the perceptions of Cardiff University medical students on the barriers faced by LGBTQ+ individuals when accessing mental health help. The study also aimed to assess recommendations Cardiff University medical students have for improvement of services and to make them more accessible to LGBTQ+ individuals.

**Methods:**

A qualitative approach was used. An online questionnaire was created and distributed among Cardiff University medical students. It included 10 open-ended questions about their knowledge of LGBTQ+ mental health, the barriers to accessing it, and recommendations to decrease these barriers. Thematic analysis was performed on the answers.

**Results:**

The questionnaire had 12 participants. The thematic analysis produced 22 subthemes, which were grouped into the following five themes: i) perceptions of LGBTQ+ mental health, ii) factors affecting LGBTQ+ mental health, iii) general barriers to accessing mental health help, iv) LGBTQ+-specific barriers to accessing mental health help, and v) recommendations to improve access to mental health services. A scoping review was conducted on this subject to further analyse the current research. The scoping review produced nine studies, and the main themes that emerged were mental health stigma, LGBTQ+ discrimination, lack of LGBTQ+-affirmative services, the pathologisation of LGBTQ+ status, and financial barriers.

**Conclusions:**

It was evident that more formal training on LGBTQ+ mental health at an undergraduate level for health professionals is needed, and that a lack of this is causing barriers to adequate provision of mental health services to this population. Furthermore, existing mental health services need to be more LGBTQ+-affirmative. Further research with a higher response rate could provide more generalisable results.

**Disclosure of Interest:**

None Declared

